# Sex-based physiological responses to a 750-m swim at critical velocity estimated by the three-parameter model

**DOI:** 10.1016/j.clinsp.2026.100943

**Published:** 2026-04-14

**Authors:** Vinícius Ribeiro dos Anjos Souza, Lavínia Vivan, Paulo Engelke, Claudio Andre Barbosa de Lira, Rodrigo Luiz Vancini, Katja Weiss, Beat Knechtle, Marilia Santos Andrade

**Affiliations:** a Post-Graduate Program in Translation Medicine, Universidade Federal de São Paulo, São Paulo, SP, Brazil; b Human and Exercise Physiology Division, Faculty of Physical Education, Universidade Federal de Goiás, Goiânia, GO, Brazil; c Center for Physical Education and Sports, Universidade Federal do Espírito Santo, Vitória, ES, Brazil; d Institute of Primary Care, University of Zurich, Zurich, Switzerland; e Medbase St. Gallen Am Vadianplatz, St. Gallen, Switzerland; f Department of Physiology, Universidade Federal de São Paulo, São Paulo, SP, Brazil

**Keywords:** Critical velocity, Swimming, Blood lactate, Sex-based difference, Triathlon

## Abstract

**Introduction:**

Critical Velocity (CV) is widely used to assess aerobic performance in endurance sports. Studies have compared protocols for CV determination in elite athletes. However, knowledge on the influence of the assessment protocol on CV and sex-based differences on metabolic responses at critical swimming velocity is limited.

**Objective:**

This study aims to compare different protocols for determining CV in swimming among male and female triathletes and to compare sex-based differences in the metabolic and perceived exertion responses after swimming 750 m at CV estimated by the three-parameter model.

**Methods:**

Highly trained amateur triathletes (12 men, 35.7 ± 11.0 years; 10 women, 38.1 ± 6.0 years) performed maximal swimming tests (50, 100, 200, and 400 m) to estimate Critical Velocity (CV). Each completed a 750 m swim at the CV determined by the three-parameter protocol (100–200–400 m), with monitoring of metabolic responses (lactate level and heart rate) and pain, dyspnea, and subjective perceived exertion.

**Results:**

The four-distance protocol yielded the highest CV in both sexes, whereas the protocol based on 92% of the 400 m time produced the lowest values (p < 0.001). After the 750 m swim, men showed higher ratings of perceived exertion (p = 0.005), pain (p < 0.001), dyspnea (p < 0.001), and blood lactate concentration (p < 0.001) than women.

**Conclusion:**

The protocol used to determine CV significantly influences estimated values. Importantly, all findings related to physiological and perceptual responses during the 750 m swim are specific to the CV determined by the three-parameter protocol and should not be generalized to other CV determination methods. Under this protocol, CV may be associated with an exercise intensity approaching the severe domain; however, this interpretation should be made with caution.

## Highlights

• Men show higher lactate, pain, dyspnea, and perceived exertion than women at CV.
• CV reflects severe-intensity domain and should not replace maximal lactate steady state.
• Critical Swimming Velocity (CV) values differ significantly by protocol choice.
• Four-distance protocol produces higher CV than two- and three-distance methods.
• 92% of 400-m test underestimates CV in male and female triathletes.

## Introduction

In 1925, Hill^1^ laid the foundation for understanding the relationship between speed and time for swimming. In 1966, Lloyd^2^ showed that the distances of world records in running increase linearly with record times. After that, the concept of Critical Velocity (CV) gained recognition as a significant performance variable among runners. Many years later, Di Prampero et al.^3^ showed that this type of analysis between velocity and time can also be applied in swimming, providing a robust framework for prescribing and evaluating swimming workouts.

CV is represented by the slope of the linear regression line when plotting time against distance.^4^^,^^5^ This line is typically constructed using data points derived from the time taken to cover various swimming distances at maximal intensity. Several previous studies have shown a strong correlation between CV and Maximal Lactate Steady State (MLSS).^6^^,^^7^ They noted no substantial differences in their measured velocities in various exercise modalities, such as running and cycling^8^, despite there not being a consensus in the literature.^9^ Lactate threshold, MLSS, or 4 mmoL/L blood lactate concentration are interesting indices for classifying training intensity domains.^10^, ^11^, ^12^ Hence, the authors can consider that CV can also be very useful in this regard to provide a basis to analyze the effects and trends brought about through training. Furthermore, CV offers the advantages of being noninvasive, cheaper, and less time-consuming. Although some studies have shown an association between Critical Velocity (CV) and the velocity corresponding to a blood lactate concentration of 4 mmoL/L^-1^, there is also evidence that these two indices are not exactly equivalent.^5^^,^^13^^,^^14^ Moreover, these findings are mostly derived from studies conducted exclusively with male swimmers, and it remains unclear whether the metabolic responses to swimming at CV are similar between male and female athletes.^5^^,^^13^^,^^14^

Regarding the protocol to determine CV, several different possibilities exist. The traditional assessment of critical swimming velocity typically requires four swimming maximal trials over distances ranging from 50 to 800 m. CV is calculated as the slope of the linear regression between Distance (D) and Time (T).^15^^,^^16^ Although widely adopted, this approach is time-consuming and physically demanding, which may limit its practicality. Consequently, alternative protocols have been proposed, including the estimation of CV from a fixed percentage of a single 400 m time-trial performance, such as 92% of the 400 m time, which has been reported as valid in trained swimmers.^4^ In parallel, studies comparing different CV determination protocols in well-trained swimmers have shown that the two-parameter model may yield higher CV values than models using three or more parameters.^10^ However, this finding is not consistently supported across the literature.^14^

In addition to the test protocol, another factor that can also impact the results of the CV test is the age and fitness level of the athletes. Previous studies demonstrated that the same test protocol can generate CV values that are either below or above the MLSS when applied to swimmers of different ages.^5^^,^^17^

The test protocol, physical fitness level, and age can influence CV. Hence, the present study mainly aimed to compare various protocols for CV determination in adult male and female highly trained amateur triathletes in swimming activity. Following the establishment of CV, a secondary aim was to compare sex-based differences in the metabolic and perceived exertion responses after swimming 750 m at the critical velocity determined by three parameters (100–200 and 400 m).^14^ The present study hypothesized that estimating CV as 92% of the maximal aerobic speed derived from a 400 m time trial would yield values comparable to those obtained using protocols with four measurements in highly trained triathletes. In addition, the authors also hypothesized that the male athletes will produce higher metabolic responses (lactate and heart rate) and subjective perceived exertion responses than female athletes after swimming 750 m at CV estimated by the three-parameter model.

## Methods

### Ethical approval

The Human Research Ethics Committee of the Federal University of São Paulo (CAAE: 66,648,122.3.0000.5505), an institution affiliated with the principal investigator, approved all experimental procedures used in this study. They were informed about the objectives, risks, and benefits of the study prior to submitting a consent form. All participants had their privacy and confidentiality guaranteed in the study.

### Participants

A total of 22 highly trained amateur triathletes (12 men and 10 women) participated in the study. They were invited through social media and sports clubs. The male group was 35 ± 11 years-old, with a body mass of 76.8 ± 6.2 kg, and measured 177.0 ± 4.2 cm. The female group was 38±6 years-old, with a body mass of 59.4 ± 5.5 kg, and measured 163.0 ± 5.6 cm. None of the female participants was in menopause. The inclusion criteria included participation in a triathlon training program for at least 1-year, between the ages of 20 and 50, and having completed the physical activity readiness questionnaire (PAR-Q).^18^ Participants who answered “no” to all questions on the PAR-Q were included in the study. The exclusion criteria included having suffered from cardiovascular, neurological, cognitive, orthopedic, and respiratory diseases assessed by the medical team.

#### Study design

This cross-sectional study was conducted in accordance with the STROBE guidelines and consisted of three visits during the morning period to the Exercise Physiology Laboratory of the Federal University of São Paulo. During visit 1, anamnesis on general health characteristics and physical activity habits was applied using a Google Form questionnaire. Participants underwent cardiopulmonary exercise testing and body composition assessment in a laboratory environment with temperatures of 19 °C–21 °C and relative humidity of 50%–60%. During visit 2, the CV test was performed. All triathletes wore a trisuit for the swim, a cap and goggles. No one wore a wetsuit. The test was performed in a 25 m pool with a water temperature of 28 °C using four measurements in descending order (400–200–100–50 m). A stopwatch (Stopwatch, HS −70 w, China) was used by an experienced and well-trained evaluator to record the time at each distance. In addition, a heart rate monitor was used to record the heart rate (Polar H10, Polar Electro, SP, Brazil), and the perceived exertion scale (Borg Scale) was applied at the end of each distance performed. Ten 10-min rest periods between each swimming distance were given. During visit 3, a 750 m swimming training session (short triathlon distance) corresponding to the CV measured using three parameters was performed. Physiological variables, maximum heart rate (Polar H10, Polar Electro, SP, Brazil), blood lactate concentration (Lactate Plus Meter Nova Biomedical, MA, EUA) were recorded at the end, including the subjective effort perception (Borg scale), painand dyspnea (visual analog scale). All triathletes also wore a trisuit for the swim, cap and goggles. Stimulants, such as caffeine or tea, were avoided for at least 8 h before each visit. In addition, participants were instructed to avoid eating within 2 h before each visit and to follow their regular hydration routine. They were also instructed not to perform physical training sessions on the days of laboratory visits.

#### Triathlon training characteristics questionnaire

Participants answered the following open-ended questions: 1) How often do you train for a triathlon? 2) How many hours do you swim per week? 3) How many hours do you cycle per week? 4) How many hours do you run per week? 5) How many hours do you do strength training per week? 6) Have you ever participated in a short course triathlon, Olympic triathlon, half Ironman (70.3), or Ironman (140.6)? and 7) How many times have you participated in each distance of these competitions?

#### Cardiopulmonary exercise testing

Cardiopulmonary exercise testing was performed under laboratory conditions on an Excalibur Sport ergometer (Lode, The Netherlands). During exercise, participants’ physiological responses (oxygen uptake, respiratory exchange rate, and heart rate) were measured using a metabolic device (Quark PFT; Cosmed, Rome, Italy). This method is a valid, accurate, and reliable tool for measuring maximal oxygen uptake (V̇O_2_max).^19^ Before each test, the equipment was calibrated according to the manufacturer’s guidelines.

All tests began with a 1-min rest period, with a 3-min warm-up period at a constant load (130 W for men and 85 W for women). The incremental protocol began with 15 W increments for men and women per stage for 1 min until voluntary exhaustion or when the cadence fell below 70 rpm.^20^ At the end of the tests, a 1-min recovery period was recorded. Pedal cadence was maintained between 70 and 90 rpm.^21^ Expiratory gas was collected breath by breath and averaged every 20 s for analysis.

The examiner verbally encouraged participants to achieve maximal performance. The Borg scale was used to determine perceived exertion at the end of each stage.^22^ The following criteria were used to identify V̇O_2_max: a plateau in the V̇O_2_ curve (increases of <150 mL/min).^23^ Moreover, the following criteria were used to identify V̇O_2_max in the absence of a V̇O_2_ plateau: respiratory exchange ratio ≥1.10, an age-predicted maximum heart rate (207–0.7 × age),^24^ and a Borg scale of perceived exertion of 20.

### Body composition assessment

The body composition (% fat mass and lean mass) of triathletes was assessed using a dual-energy X-Ray absorptiometry system (software version 12.3, Lunar DPX, Wisconsin, USA). This technique uses minimal radiation exposure and is a reliable method for assessing body composition (< 10 microSieverts).^25^

### CV swimming protocol

The CV test was performed during the morning period under controlled conditions in the official 25 m indoor pool, heated to 28 °C. The CV speed was determined from the linear relationship between the distance covered and the time required to complete it. Maximum tests in four different distances in decreasing order 400–200–100–50 m, on the same day with a 10-min rest between tests, were performed to determine the CV. Additional rest time was given if deemed necessary to return the heart rate to the rest level measured before all the tests. After performing the tests, six different mathematical models were selected to determine the critical speed (100–400), (200–400), (100–200–400), (50–100–200–400), (92%T400), and (95%T400). Notably, the 400 m all-out front crawl test (T400) is considered the maximal aerobic speed.^26^

#### Swimming test

The athletes were instructed to perform 750 m of continuous swimming at an intensity corresponding to their CV, previously determined using three reference parameters (100–200 and 400 m events). The choice of the three-parameter model was based on the study by Costa et al.,^14^ which reported that the critical velocity obtained using this protocol was not different from the velocity of 4 mmoL/L^-1^ of blood lactate concentration, although this study was conducted exclusively in men, and the results may not be generalizable to the female population. Borg perception, pain, and dyspnea scales were applied before and after the test, in addition to the heart rate and blood lactate level.

To monitor perceptual responses to effort, three validated scales were applied: the Borg scale of subjective perceived exertion (6–20 points), which quantifies the intensity of perceived effort based on the integration of physiological and psychological signals during exercise^22^; the pain scale, which measures the painful sensation resulting from muscular and metabolic stress induced by exercise^27^; and the dyspnea scale, aimed at assessing perceived respiratory difficulty due to the cardiorespiratory demands imposed by aquatic exercise.^28^ Collections were performed at rest and immediately after the end of the 750 m swimming. Blood lactate concentration was determined through a capillary sample collected from the earlobe using the portable Lactate Plus Meter analyzer (Nova Biomedical, Waltham, MA, USA). Collections were performed at rest and immediately after the end of the 750 m swimming.^29^ In addition, heart rate was monitored at rest and after the end of the swim using a heart sensor (Polar H10, Polar Electro, SP, Brazil), allowing the analysis of the cardiovascular response to effort.^30^

### Statistical analysis

The sample size was calculated using a significant effect size of 0.25, which is considered a medium effect, a significance level of 0.05, and a power of 0.80. The G*Power program (version 3.1.9.7; Heinrich-Heine-Universität Düsseldorf, Düsseldorf, Germany) was used to analyze the test power level. According to the calculations, a total sample size of 20 participants was required. Descriptive data were expressed as mean ± standard deviation, percentages, effect sizes, and statistical power. The magnitude of the observed differences was interpreted based on Cohen’s criteria: *d* < 0.2 (no effect); 0.2 ≤ *d* < 0.5 (small); 0.5 ≤ *d* < 0.8 (moderate); 0.8 ≤ *d* < 1.3 (large); and *d* ≥ 1.3 (very large).^31^ The sample size calculation (n = 22) was performed considering an effect size of 0.15, a significance level of 5% (0.05), and a statistical power of 80% (0.80). Data normality was verified using the Shapiro-Wilk test, and homogeneity of variances was assessed using the Levene test.^31^ Differences among the sexes in physical, anthropometric, performance, and physiological variables (after the 750 m swim) were analyzed using the Student’s *t*-test for independent samples. Differences in the number of participations in triathlon events between sexes were assessed using the Chi-Square test. Two-way analysis of variance for repeated measures was applied to verify the effect of the different critical swimming speed protocols and differences between sexes. Two-way analysis of variance for repeated measures was applied to verify the effect of each 100 m (of the entire 750 m swimming test) and sex on the swimming velocity. When necessary, the Sidak post hoc test was used.^31^ The significance level adopted was p < 0.05. All statistical analyses were conducted using SPSS software (version 26.0, IBM Corp., Armonk, NY, USA). All graphs were produced using GraphPad Prism (version 8.3.1, GraphPad Software Inc., La Jolla, CA, USA). Figures and Tables present all data collected in this study; raw data are available upon request to the corresponding author.

## Results

Male and female triathletes did not show statistically significant differences in terms of age, years of triathlon experience, absolute values of maximum heart rate achieved in the incremental test, and V̇O_2_max (mL/kg/min) (Table 1). Conversely, male triathletes had significantly higher total body mass, body height, and V̇O_2_max (mL/min) compared with female triathletes. In addition, female triathletes had a higher percentage of body fat than male triathletes (Table 1).

**Table 1:** Characteristics of participants.

Variables	Males Triathletes (n = 12)	Females Triathletes (n = 10)	p-value	Effect size	95% CI	Power
Age (yrs)	35.7 ± 11	38.1 ± 6	0.565	0.33	−0.52 to 1.17	0.11
Body mass (kg)	76.8 ± 6.2	59.4 ± 5.5	<0.001	2.00	0.97 to 3.03	0.99
Body height (cm)	177.2 ± 4.2	163.1 ± 5.6	<0.001	2.87	1.68 to 4.06	0.99
Body Fat %	17.3 ± 6.4	24.3 ± 7.9	<0.001	0.97	0.10 to 1.87	0.57
Experience in triathlon (yrs.)	3.3 ± 1.7	2.8 ± 1.2	0.523	0.33	0.51 to 1.18	0.11
V̇O_2_max (mL/kg/min)	50.5 ± 5.4	47.8 ± 5.2	0.259	0.50	−0.34 to 1.36	0.19
V̇O_2_max (mL/min)	3.8 ± 0.4	2.8 ± 0.3	<0.001	1.17	0.26 to 2.07	0.73
HRmax at CEPT test (bpm)	176 ± 10	176 ± 7	0.927	0.00	−0.84 to 0.84	0.05

*Note*: yrs, Years; kg, Kkilogram; cm, Centimeters; BF%, Body Fat; CEPT, Cardiopulmonary Exercise Testing; V̇O_2_max, Maximal Oxygen uptake; HR, Heart Hate; bpm, Beats per minute; Effect size, *Cohen’s d*; CI, Confidence Interval; power, 1−*β*.

Data are presented as mean ± standard deviation.

No significant differences were identified between male and female triathletes in terms of weekly training volume, either in kilometers or hours, in the swimming, cycling, running, and strength training modalities (Table 2). All participants reported previous experience in multiple triathlon competitions. In addition, no differences were observed between sexes in terms of the number of previous participations in each of the modality’s distances (Table 2).

**Table 2:** Training characteristics of the participants.

Variables	Males Triathletes (n = 12)	Females Triathletes (n = 10)	p-value	Effect size	95% CI	Power
Swimming (min per week)	157 ± 64	157 ± 70	0.986	0.00	−0.84 to 0.84	0.05
Cycling (min per week)	340 ± 162	260 ± 65	0.161	0.63	−0.23 to 1.49	0.28
Running (min per week)	232 ± 55	200 ± 67	0.229	0.53	−0.33 to 1.38	0.21
Swimming (km per week)	7.0 ± 2.3	5.1 ± 2.5	0.084	0.84	−0.04 to 1.71	0.46
Cycling (km per week)	168 ± 89	127 ± 46	0209	0.56	−0.29 to 1.42	0.23
Running (km per week)	43.6 ± 14.9	30.5 ± 12.0	0.037	0.96	0.07 to 1.84	0.56
Strength training (frequency/week)	2 ± 1	2 ± 1	0.797	0.00	−0.84 to 0.84	0.05
Participation in triathlon events	Yes	Yes				
Short triathlon	10 (83%)	8 (80%)	0.500	‒	‒	‒
Best time for hh:min:ss	1:19:00 ± 0.15:55	1:23:33 ± 0:09:39	0.553	0.32	−0.53 to 1.16	0.10
Triathlon Olympic	9 (75%)	5 (50%)	0.110	‒	‒	‒
Best time for hh:min:ss	2:23:21 ± 0:16:14	2:39:00 ± 0:11:00	0.076	1.15	0.24 to 2.05	0.72
Half Ironman (70.3)	7 (58%)	4 (40%)	0.137	‒	‒	‒
Best time for hh:min:ss	5:18:56 ± 1:30:37	5:54:48 ± 0:18:19	0.227	0.37	−0.48 to 1.22	0.13
Ironman	2 (16%)	‒	‒	‒	‒	‒
Best time for hh:min:ss	9:48:30 ± 0:44:32	‒	‒	‒	‒	‒

*Note*: hh, Hours; min, Minute; ss, Seconds; km, Kilometers. Data are presented as mean ± standard deviation. Effect size, *Cohen’s d*; CI, Confidence Interval; power, 1−*β*.

Table 3 presents the total times, in seconds, obtained in the different swimming distances (50, 100, 200, and 400 m) for each sex. Male triathletes presented significantly lower times in all distances analyzed (p < 0.005) than female triathletes.

**Table 3:** Mean values and Standard Deviations (±SD) of critical speed (s) obtained over distances of 50–400 m for male and female triathletes.

Variables	Males Triathletes (n = 12)	Females Triathletes (n = 10)	p-value	Effect size	95% CI	Power
50 m (s)	38.17 ± 4.14	48.47 ± 9.50	0.003	1.46	0.51 to 2.40	0.90
100 m (s)	86.76 ± 10.4	107.21 ± 22.5	0.011	1.21	0.29 to 2.12	0.76
200 m (s)	190.76 ± 23.6	232.59 ± 49.5	0.017	1.11	0.21 to 2.02	0.69
400 m (s)	400.16 ± 47.4	477.93 ± 91.3	0.018	1.10	0.20 to 2.00	0.68

*Note*: m, Meters; s, Seconds; Effect size, *Eta squared*; CI, Confidence Interval; power, 1−*β*. Data are presented as mean ± standard deviation.

**Fig. 1:**
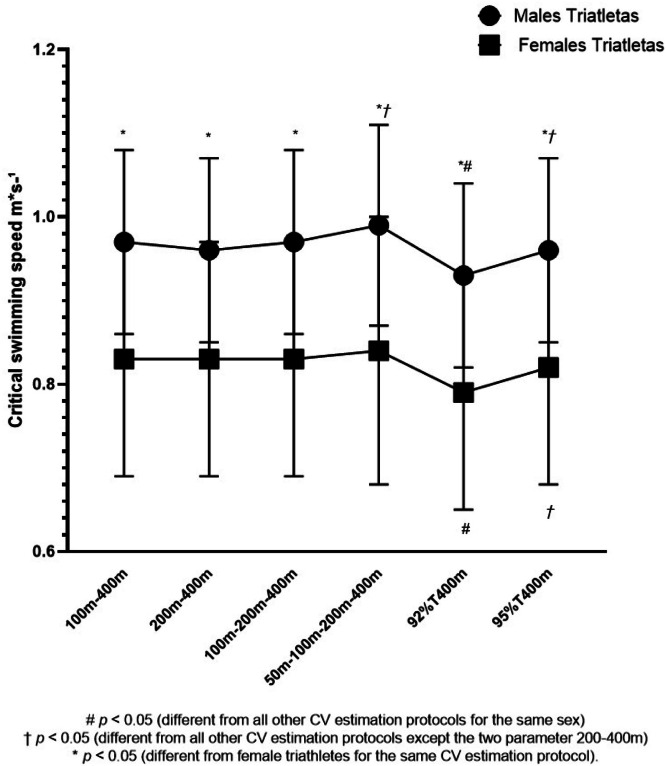
Comparison of different protocols for determining critical velocity in men and women.

Regarding the comparison among different protocols to determine CV and between sexes, the present data indicate a significant sex effect [F(1; 20) = 6.163; p = 0.022; effect size 0.23; power = 0.23], and protocol effect [F(1; 20) = 51.678; p < 0.001; effect size 0.72; power = 1.0], however there are no interaction effect (sex × protocol) [F(1; 20) = 1.621; p = 0.215; effect size 0.07; power = 0.28]. In male triathletes, the protocol with four measurements presented significantly higher values than the protocol with three measurements (p < 0.001) and two measurements, 100–400 (p = 0.004), 92% of the 400 m time (p < 0.001), and 95% of the 400 m time (p < 0.001). The protocol using 92% of the 400 m time presented significantly lower values among all other protocols (p < 0.001). Finally, the protocol using 95% of the 400 m time presented significantly different values than the protocol with three measurements (p = 0.005) and two measurements 100–400 (p = 0.022), with four measurements (p < 0.001) and 92% of the 400 m time (p < 0.001) (Table 4 and Fig. 1).

**Table 4:** Comparison of different critical velocity estimation protocols between male and female triathletes.

Variables	Male Triathletes (n = 12)	Female Triathletes (n = 10)
Two-parameter model (100‒400) (m.s^-1^)	0.97 ± 0.12^a^	0.82 ± 0.12
Two-parameter model (200‒400) (m.s^-1^)	0.97 ± 0.12^a^	0.82 ± 0.11
Three-parameter model (100‒200‒400) (m.s^-1^)	0.97 ± 0.12^a^	0.82 ± 0.12
Four-parameter model (50‒100‒200‒400) (m.s^-1^)	1.00 ± 0.13^a^^,^^b^	0.83 ± 0.13
92% Time 400 m parameter model (m.s^-1^)	0.93 ± 0.11^a^^,^^c^	0.78 ± 0.11^c^
95% Time 400 m parameter model (m.s^-1^)	0.96 ± 0.12^a^^,^^b^	0.80 ± 0.12^b^

Data are presented as mean ± standard deviation.

a p < 0.05 (different from female triathletes for the same CV estimation protocol).

b p < 0.05 (different from all other CV estimation protocols except the two parameter 200‒400 m).

c p < 0.05 (different from all other CV estimation protocols for the same sex).

In female triathletes, the protocol with four measurements presented significantly higher values than the protocols using 92% of the 400 m time (p < 0.001) and 95% of the 400 m time (p < 0.001). The protocol using 92% of the 400 m time presented significantly lower values among all other protocols (p < 0.001). Finally, the protocol using 95% of the 400 m time presented significantly different values than the protocol with three measurements (p = 0.004) and two measurements 100–400 (p = 0.001), with four measurements (p = 0.009) and 92% of the 400 m time (p < 0.001) (Table 4 and Fig. 1).

**Fig. 2:**
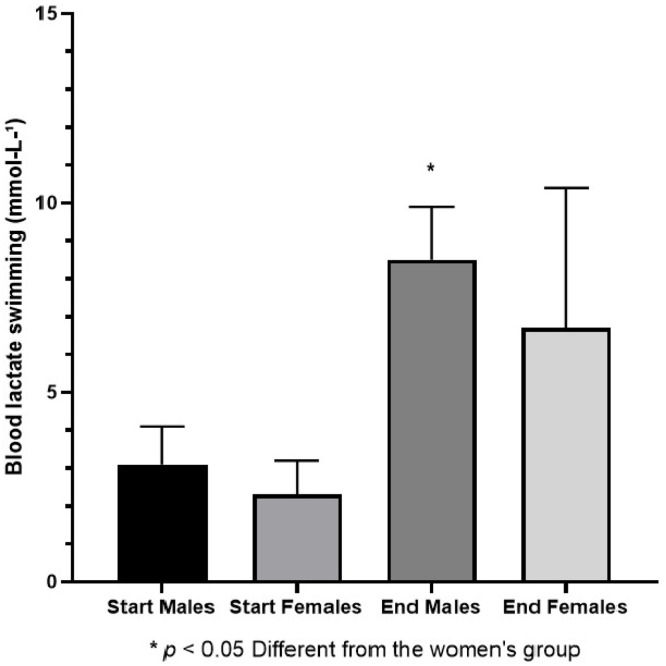
Blood lactate values measured before and after the 750 m swimming test in men and women.

After the analysis of the critical speed determination protocols, all triathletes swam 750 m at the critical speed determined by three parameters (100–200 and 400 m). Men swam at 1.00 ± 0.11 m/s, and the mean critical speed was 0.99 ± 0.12 m/s (p = 0.547). Women swam at 0.83 ± 0.14 m/s, and the mean critical swimming speed was 0.84 ± 0.13 m/s (p = 0.431). To examine pacing maintenance throughout the 750 m swim in male and female athletes, the authors compared the velocity sustained in each 100 m segment for both sexes. The results showed a significant effect of distance [F(7112) = 55.77; p < 0.001], no effect of sex [F(1, 16) = 4.33; p = 0.054], and a significant interaction effect [F(7112) = 5.18; p < 0.001]. Post hoc analysis showed that women swam the first 100 m faster than the second 100 m (p = 0.006), after which their pace remained stable, with no further differences up to the end of the 750 m (p = 0.725). Similarly, men swam the first (p < 0.01) and second 100 m (p = 0.005) faster than the third 100 m, after which their pace stabilized, with no significant differences thereafter (p = 0.680). The rest values for perception of effort, pain, dyspnea, and heart rate did not show statistically significant differences between sexes (p > 0.005). After a 750 m swim, male triathletes showed significantly higher values than female triathletes in terms of perception of effort (p = 0.005), pain (p < 0.001), dyspnea (p < 0.001), lactate level (p < 0.001), and similar heart rate (p = 0.075) (Table 5 and Fig. 2). Despite no difference in heart rate between sexes at the end of the 750 m swim, men finished at 85% ± 7% of HRmax, whereas women finished at 81% ± 5% of HRmax.

**Table 5:** Metabolic and subjective responses after 750 m of swimming at Critical Velocity Estimated by the Three-Parameter Model.

Variables	Males Triathletes (n = 10)	Females Triathletes (n = 9)	p-value	Effect size	95% CI	Power
**Baseline effort perception (6‒20)**	6.9 ± 0.9	6.9 ± 1.3	0.845	0.00	−0.90 to 0.90	0.05
**Final effort perception (6‒20)**	17.1 ± 2.6^a^	15.5 ± 2.7	0.005	0.60	−0.32 to 1.53	0.34
**Baseline pain (0‒10)**	1.0 ± 1.2	2.4 ± 1.6	0.668	1.00	0.04 to 1.95	0.67
**Final pain (0‒10)**	5.6 ± 1.0^a^	4.0 ± 2.3	<0.001	0.92	−0.03 to 1.87	0.60
**Baseline dyspnea (0‒10)**	0.3 ± 0.5	0.8 ± 0.8	0.504	0.76	−0.17 to 1.69	0.47
**Final dyspnea (0‒10)**	7.1 ± 1.9^a^	5.5 ± 1.7	<0.001	0.88	−0,06 to 1,83	0.57
**Baseline blood lactate (mmoL/L^-1^)**	3.1 ± 1.0	2.3 ± 0.9	0.119	0.84	−0.10 to 1.78	0.54
**Final blood lactate (mmoL/L^-1^)**	8.5 ± 1.4^a^	6.7 ± 3.7	<0.001	0.66	−0.24 to 1.58	0.39
**Baseline heart rate (bpm)**	84 ± 13	86 ± 11	0.865	0.17	−0.74 to 1.07	0.09
**Final heart rate (bpm)**	150 ± 15	143 ± 15	0.075	0.47	−0.45 to 1.38	0.25

*Note*: Effect size, *Cohen’s d*; CI, Confidence Interval; power, 1−*β*. Data are presented as mean ± standard deviation.

a Different from the group female.

## Discussion

This study compared different protocols to determine critical swimming speed among male and female triathletes and to evaluate the physiological responses to performing 750 m of swimming at VC with the hypotheses of determining CV at 92% of the maximum aerobic speed of the 400-meter time could be similar to the protocols performed with four measurements. In addition, it was also hypothesized that the male athletes will produce higher metabolic responses (lactate and heart rate) and subjective perceived exertion responses than female athletes. The main findings of this study were the following: a) The protocol to determine CV by 92% of T-400 m resulted in the lowest CV values in both sexes. b) The protocol to determine CV using four parameters resulted in higher CV values. c) The protocol effect to determine CV was similar between sexes. d) Swimming 750 m (mean time for male and female athletes) at CV estimated by the three-parameter model produces a higher metabolic stress than the estimated for MLSS. e) Male athletes experienced higher metabolic stress than females when swimming 750 m at CV estimated by the three-parameter model. The hypothesis of this study was partially confirmed as the protocols used to determine CV using four parameters resulted in higher CV values, whereas protocols using 92% of the T-400 m resulted in the lowest values. Furthermore, men showed higher lactate values when swimming 750 m at CV estimated by the three-parameter model than women, indicating greater metabolic stress.

This study, developed among highly trained amateur triathletes, demonstrated that the protocol used to estimate CV significantly influences the results. The four-parameter protocol produced significantly higher CV than the other protocols for male and female athletes. Other authors have extensively used the four-parameter protocol, which commonly involves the 50 m distance.^32^ However, the CV test was originally recommended to be performed with series lasting between 2 and 15 min.^11^ This distance may be too short and has a predominance of anaerobic metabolism because it can be completed in <1 min.^33^ This case could compromise the CV test because overestimating its value may not adequately represent the maximal sustainable aerobic swimming speed.^4^ However, the literature has not reached a consensus on these findings; a similar CV calculated from combinations of four distances has once presented similar results to the CV calculated from two or three parameters.^10^ These seemingly different results were produced possibly because of the varying characteristic CVs of the studied population. Toubekis et al.^5^ studied young swimmers, whereas the present study examined adult amateur triathletes. In addition, age and fitness level have been described as potential factors that can affect the results obtained from the CV test.

In addition to calculating CV using four-, three-, and two-parameter models, the present study also estimated CV based on fixed percentages (95% and 92%) of the 400 m time trial (T-400 m). The use of 92% of T-400 m has been previously proposed as a practical alternative for CV determination,^4^^,^^32^^,^^34^ as it requires only a single 400 m test rather than multiple exhaustive swimming trials, which may be particularly appealing to swimming coaches. However, in the present study, CV values estimated using 92% of the T-400 m were systematically lower than those obtained with the two- and three-parameter models. This finding contrasts with earlier reports suggesting equivalence between CV derived from 92% of T-400 m and four-parameter models,^4^ and may be explained by differences in participant characteristics, including age, sex distribution, and training status. Importantly, the significant differences observed among the protocols (F = 51.68; p < 0.001) underscore the need for careful selection of the method used to determine critical velocity, as inaccuracies in CV estimation may directly affect training-load monitoring and exercise intensity prescription in endurance training.^35^

Another very interesting finding of this study was that the differences among the test protocols for determining CV were similar between sexes because no interaction effect was observed between sex and protocol. This finding is quite important because most previous studies, despite having evaluated both sexes, did not compare them.^4^^,^^32^ In addition, men presented significantly higher critical speed values compared with women, regardless of the protocol adopted. This result reflects known physiological differences between sexes, such as greater cardiorespiratory capacity, greater muscle mass, and lower body fat percentage in men.^36^

The second part of the study evaluated the physiological and subjective responses after swimming 750 m at the determined CV estimated by three-parameter model. Regarding swimming speed maintained during the 750 m test, the results showed that it did not differ from the critical velocity estimated by the three-parameter model. In addition to average speed, the authors also assessed pacing throughout the 750 m by comparing the time for each 100 m segment. The authors found that, in the first 100 m, both men and women swam faster, as indicated by shorter split times. However, after this initial segment, the times remained stable, indicating that swimmers maintained a consistent pace throughout the remainder of the test. Men and women maintained similar swimming speeds to the CV. However, despite both sexes swimming at CV estimated by the three-parameter model, male triathletes presented significantly higher lactate levels, pain, dyspnea, and effort perception than female athletes at the end of the 750 m swim. Although no statistically significant difference was found in heart rate at the end of the 750 m swim, the statistical power for this comparison was low (power = 0.25). This limitation substantially increases the potential for a Type II error, indicating that a true sex-based difference in heart rate may be present but not detected by the analysis. Therefore, non-significant findings for this variable should be interpreted with particular caution. Furthermore, the male group finished with a mean HR of 150 ± 15 bpm and the female group with 143 ± 15 bpm, corresponding to 85% ± 7% and 81% ± 5% of their respective maximal heart rates, respectively. When heart rate is expressed relative to individual maximal values, these differences suggest a higher relative cardiovascular strain in men, as exercising at a greater percentage of HRmax reflects a higher cardiac demand to sustain the imposed workload. Taken together, these findings support the interpretation that the overall physiological strain during the 750 m swim was greater in men than in women, despite the absence of statistical significance in absolute heart rate values. It is also noted previously that the female athletes presented lower lactate levels while performing at similar relative intensities, which may indicate a more efficient lactate clearance or utilization.^37^ Moreover, a superior oxidative capacity, including the lower muscle mass compared with men,^38^ may be associated with these lower blood lactate levels and reduced ratings of perceived exertion. Nevertheless, these interpretations remain speculative, and further studies are warranted to systematically test these hypotheses and elucidate the underlying physiological mechanisms. Moreover, the lactate level at the end of the 750 m swimming at CV estimated by the three-parameter model was 8.5 ± 1.4 for male and 6.7 ± 3.7 for female athletes. Although these values are relatively high, they should be interpreted with caution, as post-exercise lactate concentrations alone do not allow definitive classification of the exercise intensity domain. Thus, these findings may suggest that continuous swimming at a CV determined by the three-parameter model could be associated with intensities approaching the severe domain; however, this interpretation remains speculative in the absence of direct MLSS assessment. These data are in accordance with previous literature data.^39^ The determination of CV leads to an overestimation of the metabolic rate associated with MLSS.^40^ Therefore, even if CV is strongly correlated with MLSS, these two variables are different, indicating that CV estimated by three parameters and MLSS are different and are not necessarily interchangeable. However, the literature has not reached a consensus because other studies supported the notion that swimming at CV corresponds to the MLSS.^41^ Possibly, the large difference in fitness levels among the participants might be associated with these results. Takahashi et al. (2009) studied highly trained swimmers, whereas in the present study, the authors evaluated amateur triathletes. Moreover, there is likely a substantial difference in swimming technique and, consequently, in swimming economy. Takahashi et al.^41^ evaluated swimmers with more than nine years of experience in competitive swimming, whereas the participants were amateur triathletes with considerably less sporting experience. The correspondence between MLSS and CV is perhaps only true for very well-trained athletes in the specific sport in question (swimming). However, future studies should be developed comparing both tests among athletes presenting different physical conditioning levels to elucidate this point.

### Study limitations and strengths

The strengths of the study design include the analysis of data from male and female triathletes separately, allowing for different conclusions to be drawn for each sex. In addition, the study design included different critical speed protocols. The study was also developed with highly trained amateur triathletes; however, additional studies with professional or recreational triathletes may yield different results. Therefore, conducting additional studies involving professional or recreational triathletes is crucial. Conversely, the absence of mechanistic data that could help explain the sex-based differences observed limits the authors’ ability to interpret the underlying physiological determinants of these findings. Moreover, it is important to emphasize that, in the present study, critical velocity was determined using the three-velocity protocol, which also served as the basis for assessing the metabolic and perceptual responses to swimming performed at this intensity. The selection of the three-parameter model was based on Costa et al. (2009), who reported that the critical velocity estimated by this protocol did not differ from the velocity corresponding to 4 mmoL/L^-1^ of blood lactate. However, because that study included only male participants, its applicability to female triathletes may be limited. In this direction, the use of an alternative protocol for determining critical velocity could potentially elicit different metabolic and perceptual responses, highlighting the methodological dependence of the observed outcomes. Another limitation of the study is the low statistical power observed in some sex-based comparisons, which increases the risk of Type II error and limits the ability to detect true differences between men and women. Consequently, the absence of statistically significant sex differences for certain variables, such as maximal heart rate after the 750 m swim, should be interpreted with caution.

### Implications for future research

Future research should explore the applicability of different critical speed protocols across varying triathlon distances, as well as in long-term training contexts. Furthermore, further investigation is needed to clarify the physiological mechanisms that explain sex differences in metabolic and perceptual responses to effort at intensities near CV.

### Practical applications

This study contributes to the optimization of swimming intensity prescription in triathletes, highlighting that choosing a protocol for assessing critical speed and the physiological particularities related to sex is important. These findings have direct implications for load planning in endurance sports, such as triathlon. Furthermore, the findings reinforce the need for individualized approaches to maximize performance and minimize the risk of excessive fatigue.

## Conclusion

The results of this study demonstrate that the choice of evaluation protocol significantly affects the estimated critical swimming speed in male and female triathletes. However, when considering only the three-parameter protocol (100, 200, and 400 m), physiological responses during the 750 m swim tend to be more pronounced in men than in women, including higher blood lactate concentrations, greater perceived exertion, and increased physical discomfort. This result highlights potential differences in adaptations to effort that should be considered when individualizing training. Importantly, the findings of the present study should be interpreted as being specific to the critical velocity estimated using the three-parameter protocol, and caution is warranted when extrapolating these results to other CV determination methods. Finally, when determined by three parameters, CV may correspond to an intensity within the severe domain; however, although post-exercise blood lactate concentrations were high, this measure alone does not definitively classify the intensity domain. Moreover, because no direct comparison with MLSS was performed, this interpretation should be made with caution.

## Ethics approval

All experimental procedures were approved by the human research ethics committee of the relevant institution and adhered to the principles outlined in the Declaration of Helsinki. The study was approved by the Research Ethics Committee of the Federal University of São Paulo under number 7.351.985.

## Clinical trial number

The study was registered in ReBEC ‒ Registro Brasileiro de Ensaios Clinicos: Rio de Janeiro (RJ): Instituto de Informação Científica e Tecnológica em Saúde (Brazil); 2010 ‒ Identifier RBR-73vcyff. Available from http://ensaiosclinicos.gov.br/rg/RBR-73vcyff.

## Consent to participate

All study participants provided written informed consent.

## Data availability

Data supporting the study results can be provided, followed by a request sent to the corresponding author’s e-mail.

## Authors’ contributions

Conceptualization, L.V. and M.A.; Methodology, L.V., V.R.A.S., P.E.; Validation, M.S.A. and L.V.; Formal Analysis, L.V.; Investigation, L.V.; Resources, M.S.A.; Data Curation, L.V., V.R.A.S., P.E.; Writing-original Draft Preparation, L.V., M.S.A., C.A.B.L.; Writing-review & Editing, K.W., B.K., R.L.V, C.A.B.L.; Visualization, P.N; Supervision and Project Administration, M.S.A.

## Funding

This work was supported by the Fundação de Amparo à Pesquisa do Estado de São Paulo (FAPESP) (grant number: 2021/08114–1).

## Declaration of competing interest

The authors have no conflicts of interest to declare relevant to the content of this article.
